# Evaluation of the Genotoxic and Oxidative Damage Potential of Silver Nanoparticles in Human NCM460 and HCT116 Cells

**DOI:** 10.3390/ijms21051618

**Published:** 2020-02-27

**Authors:** Mingxi Jia, Wenjing Zhang, Taojin He, Meng Shu, Jing Deng, Jianhui Wang, Wen Li, Jie Bai, Qinlu Lin, Feijun Luo, Wenhua Zhou, Xiaoxi Zeng

**Affiliations:** 1 Hunan Key Laboratory of Processed Food for Special Medical Purpose, Hunan Key Laboratory of Grain-Oil Deep Process and Quality Control, College of Food Science and Engineering, National Engineering Laboratory for Deep Process of Rice and Byproducts, Central South University of Forestry and Technology, Changsha 410004, China; minxijia@outlook.com (M.J.); zswenjing@foxmail.com (W.Z.); hetaojin@outlook.com (T.H.); Shu-Meng@outlook.com (M.S.); dengjing0102@yahoo.com (J.D.); LinQL0403@126.com (Q.L.); luofeijun@outlook.com (F.L.); zhouwenhua@outlook.com (W.Z.); 2 Key Laboratory of Biological Nanomaterials and Devices, College of life sciences and chemistry, Hunan University of Technology, Zhuzhou 412007, China; zengxiaoxi2003@hut.edu.cn; 3 School of Chemistry and Food Engineering, Changsha University of Science and Technology, Changsha 410114, China; wangjh0909@csust.edu.cn

**Keywords:** nano-Ag, colon cells, biological toxicity, oxidative damage

## Abstract

Nano Ag has excellent antibacterial properties and is widely used in various antibacterial materials, such as antibacterial medicine and medical devices, food packaging materials and antibacterial textiles. Despite the many benefits of nano-Ag, more and more research indicates that it may have potential biotoxic effects. Studies have shown that people who ingest nanoparticles by mouth have the highest uptake in the intestinal tract, and that the colon area is the most vulnerable to damage and causes the disease. In this study, we examined the toxic effects of different concentrations of Ag-NPs on normal human colon cells (NCM460) and human colon cancer cells (HCT116). As the concentration of nanoparticles increased, the activity of the two colon cells decreased and intracellular reactive oxygen species (ROS) increased. RT-qPCR and Western-blot analyses showed that Ag NPs can promote the increase in P38 protein phosphorylation levels in two colon cells and promote the expression of P53 and Bax. The analysis also showed that Ag NPs can promote the down-regulation of Bcl-2, leading to an increased Bax/Bcl-2 ratio and activation of P21, further accelerating cell death. This study showed that a low concentration of nano Ag has no obvious toxic effect on colon cells, while nano Ag with concentrations higher than 15 μg/mL will cause oxidative damage to colon cells.

## 1. Introduction

Nanomaterials have unique physical and chemical properties and are widely used in medical equipment, industrial fields, biomedical fields, electronics and environmental research [1]. Especially in recent years, their use in medicine, health products and food has grown exponentially [2]. Human exposure to nanomaterials in daily life is increasing rapidly, so any potential harmful health effects need special attention.

Nano-Ag is one of the most widely used materials in the world, used in everyday consumer products [3]. Silver nanoparticles (Ag NPs) have highly effective antibacterial properties and have been widely used in medicine, medical devices, coatings, textiles, food and cosmetics to inhibit bacterial growth [4,5,6,7]. In contrast to the widespread use of nanomaterials, their biological and toxicological information is still relatively lacking. It is well known that the physical and chemical characteristics of NPs are the key factors affecting their biological effects [8]. Compared with conventional materials, the highly specific surface area of Ag-NPs enhances their interaction with components such as serum, saliva, mucus and lung lining fluid, which may cause negative effects on biological systems [9]. On the other hand, Ag-NPs can also cause strong oxidative activity by releasing Ag^+^, and can then induce cytotoxicity, genotoxicity, immune responses and even cell death [10,11,12].

The transport and distribution of nanoparticles in the body depends mainly on the blood circulation system. Nanoparticles can destroy lung epithelial cells, intestinal epithelial cells and vascular endothelial cells, resulting in increased cell membrane permeability. This allows nanoparticles to easily enter blood vessels and be transported throughout the body [13]. Although no significant human disease has been attributed to food-grade nanoparticles to date, existing research suggests that NPs may cause potentially adverse biological reactions. Nanoparticles can interact with various components of the coagulation system in blood vessels and regulate their activity [14]. For example, Ag-NPs can induce platelet aggregation and enhance thrombus formation in rats [15], and silver colloids can cause platelet aggregation and fibrin polymerization [16]. Further, nanoparticles are distributed to tissues and organs such as the liver, kidneys, brain, testis and ovaries through blood circulation. This distribution can induce the production of reactive oxygen species (ROS), causing cellular oxidative damage, immune response and genetic damage [17,18,19].

At present, little is known about the multiple mechanisms of action of Ag-NPs biological toxicity and their physiological effects on humans during short-term exposure [20,21]. The toxicity of other nanoparticles in different organisms has been reported in many studies, but the toxicity of Ag-NPs has not been widely explored, especially the colonic cytotoxicity of Ag-NPs. Studies have shown that people who ingest nanoparticles by mouth have the highest uptake in the intestinal tract, and that the colon area is the most vulnerable to damage and causes the disease. In this study, we used normal human colon cells (NCM460) and human colon cancer cells (HCT116) as models to detect cytotoxicity, oxidative stress and apoptosis after exposure to Ag-NPs and to investigate the molecular mechanisms involved.

## 2. Results

### 2.1. Characterization of Ag-NPs

**Figure 1:**
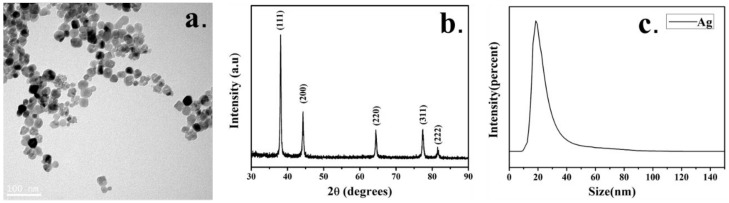
Characterization of nano-Ag. (**a**) Transmission electron micrograph; (**b**) XRD pattern. (**c**) Granularity analysis.

The characteristics of the Ag-NPs used in this study are shown in Figure 1. The TEM pattern shows that the nanoparticles have good dispersibility, a uniform size and a complete morphology. A particle size analysis shows that the size of the nanoparticles is mainly distributed around 20 nm. The XRD pattern of nano-Ag is basically the same as that of the standard JCPDS card (JCPDS NO14-0781). The characteristic peaks are shown in Figure 1b. The figure shows five obvious peaks, indicating that the nano-Ag has five crystal planes, and the corresponding indices are (111), (200), (220), (311), (322) from inside to outside. This is because there are a large number of finely oriented crystal particles in the sample area irradiated by the electron incident beam, which belongs to a polycrystalline structure.

### 2.2. Morphology Changes of Ag-NPs Treated Cells

**Figure 2:**
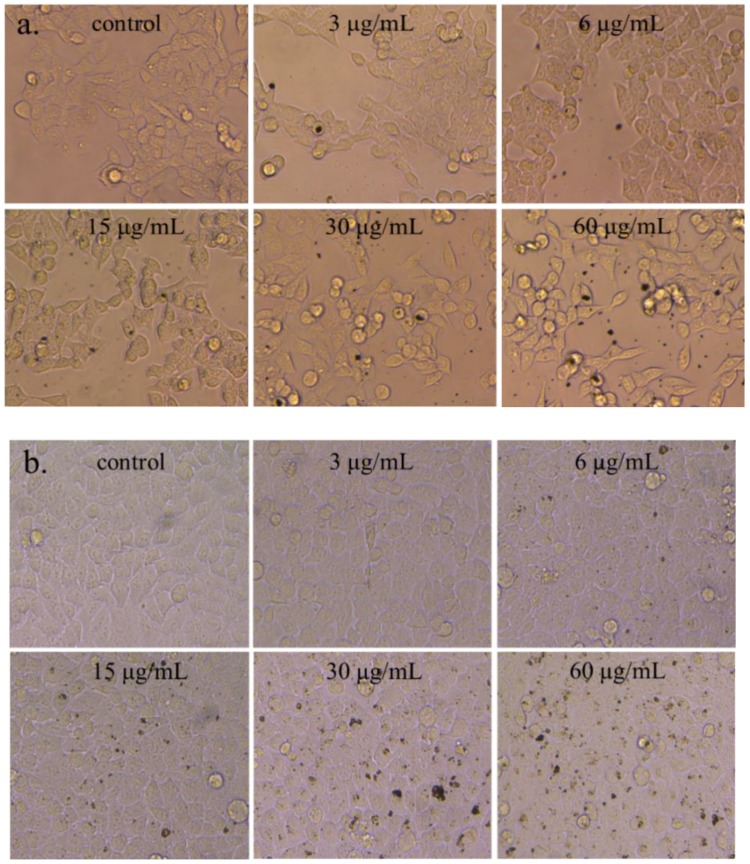
The morphology of two cells was observed after exposure to nano-Ag. (**a**) HCT116 cells; (**b**) NCM460 cells.

After the cells were exposed to different concentrations of Ag-NPs for 24 h, the cell morphology was observed through an inverted microscope, as shown in Figure 2. When the Ag-NPs concentration was 3–6 μg/mL, there was no significant change in cell morphology. When the Ag-NPs concentration was higher than 15 μg/mL, the morphology of the two colon cells changed, the cells became round and the intercellular space increased.

### 2.3. Cytotoxicity Analysis

**Figure 3:**
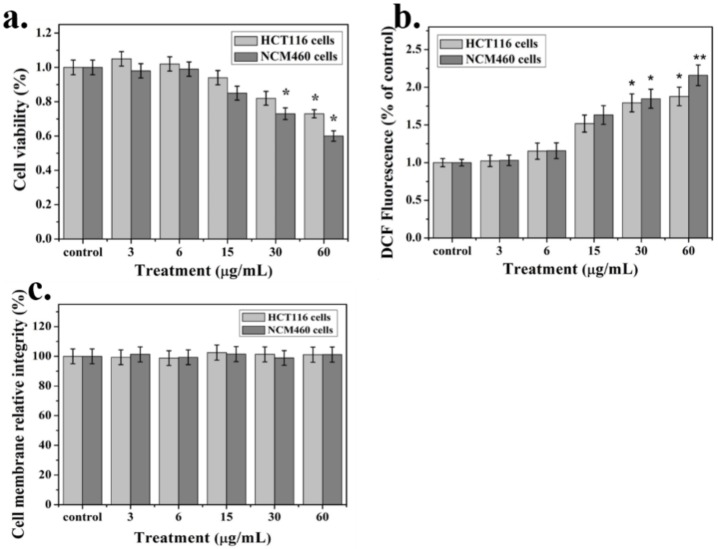
Nano-Ag cytotoxicity test. (**a**) MTT assay for cell viability; (**b**) Intracellular ROS content after exposure to nano Ag; (**c**) The lactate dehydrogenase (LDH) content in the medium was measured to assess the integrity of the cell membrane.

**Figure 4:**
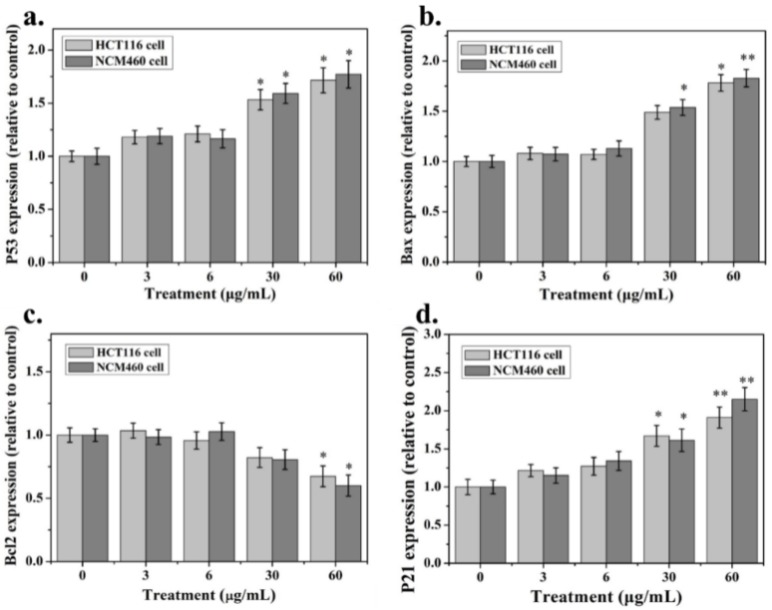
RT-PCR Analysis of the Expression Changes in mRNA Level of P53, Bax, Bcl-2, P21 after exposure to Ag NPs for HCT116 and NCM460 cells. The experiment was in triplicate and data represent mean. * means *p* < 0.05; ** means *p* < 0.01.

The cellular activity of the two colon cells after exposure to different concentrations of nanoparticles is shown in Figure 3a. After 24 h of exposure to Ag-NPs at a concentration of 3–6 μg/mL, the activity of the two cells did not change significantly. As the nanoparticle concentration increased, the viability of the two cells began to decrease when it was higher than 15 μg/mL. When the nanoparticle concentration was 60 μg/mL, the viability of NCM460 cells decreased to 60%, about 15% lower than that of the HCT116 cells. Ag-NPs showed toxicity to colon cells and inhibited cell viability at 15–60 μg/mL. A flow cytometry analysis of HCT116 cells showed that with the increase in nano Ag concentration, the proportion of apoptosis increased (Supplementary Figure S1). We also examined the levels of ROS in cells exposed to different concentrations of Ag NPs (Figure 4). When the nanoparticle concentration was 15 μg/mL, the ROS content increased significantly by about 50%. When the nanoparticle concentration reached 60 μg/mL, the intracellular ROS content increased by a factor of 2 relative to the control group (Figure 3b). However, within the tested nanoparticle concentration, there was no significant difference in the lactate dehydrogenase (LDH) concentration in the medium of each experimental group, indicating that the integrity of the cell membrane was not damaged (Figure 3c).

### 2.4. Apoptosis-Associated mRNA and Protein Expression

The mRNA levels of the cell apoptosis markers (P53, Bax, Bcl-2, P21 and GAPDH) were detected by RT-PCR after exposure to Ag-NPs and are shown in Figure 4. After exposure to 3 and 6 μg/mL Ag NPs, the expression of P53, Bax, Bcl-2 and P21 did not change significantly in the two colon cells. When the nanoparticle concentration reached 30–60 μg/mL, P53, Bax and P21 significantly up-regulated expression, while Bcl-2 down-regulated expression.

**Figure 5:**
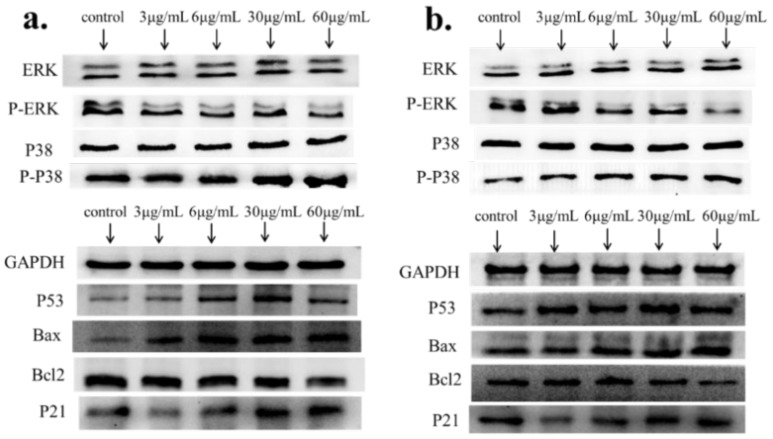
Western-blot analysis of the expression changes in protein level of P53, Bax, Bcl-2, P21 after exposure to Ag NPs for HCT116 and NCM460 cells. (**a**) HCT116 cell protein expression result; (**b**) NCM460 cell protein expression result.

Further, we performed Western-blot on the protein expression of related genes (Figure 5). In HCT116 cells, when the nano Ag concentration was 3–6 μg/mL, there was no significant change in the expression of each related protein. When the nano-Ag concentration was increased to 30 μg/mL, the phosphorylation level of the P38 protein increased, but the phosphorylation level of the ERK protein did not change significantly. At the same time, the expression of the P53, Bax and P21 proteins increased and the expression of Bcl-2 decreased. In the NCM460 cells, the expression of the P38, P53, Bax and P21 proteins was the same as that of the HCT116 cells, with the difference being that the phosphorylation level of the ERK protein was reduced. The results of Western-blot were consistent with mRNA expression.

## 3. Discussion

Currently, Ag NPs are widely used as antibacterial agents in various foods, cosmetics, healthcare products and medical devices. With the increasing application of Ag NPs in daily life, their toxicology and environmental impact have caused widespread concern. No matter how the nanoparticles enter the human body, they can reach all parts of the body through the blood system. Due to the special size of the nanoparticles, they may remain in human organs and cause toxic effects. In vivo studies have shown that the accumulation of Ag NPs in organs may cause pathological changes in organs such as the liver, kidneys and lungs [22,23]. In recent years, research on the biological safety of nanoparticles has become a hot topic. The cytotoxicity of many nanoparticles related to food, cosmetics and medicine, such as Ag, TiO_2_ and ZnO [24,25,26], has been studied. Studies have shown that Ag NPs can induce cytotoxicity and genotoxicity in human cells [27]. At present, the interaction between Ag NPs and mammalian systems is not fully understood and the underlying mechanisms of Ag NPs toxicity are poorly understood.

In this study, HCT116 and NCM460 cells were selected to evaluate the colonic cytotoxicity of nano Ag. Morphological observation is the easiest and most direct way to identify cells, because the morphology of most cells is related to the action of drugs or poisons. After treatment with Ag NPs, the shape of the two colon cells changed, while the controls remained unchanged. Cell viability testing is a vital step in toxicology testing which shows the response of cells to drugs and provides information about cell death and survival. In this experiment, Ag NPs did not significantly inhibit the growth of the two cells in 0–6 μg/mL. As the nanoparticle concentration increased, the viability of the two cells began to decrease when it was higher than 15 μg/mL. When the nanoparticle concentration was 60 μg/mL, the viability of NCM460 cells decreased to 60%, which was about 15% lower than that of HCT116 cells. These results indicate that the cytotoxicity of Ag NP is positively correlated with its concentration. In addition, previous studies have shown that Ag NPs can release Ag ^+^ in biological fluids, while cytotoxicity is largely caused by Ag^+^ [28]. This will be further studied in our next work.

The ROS mechanism is one of the most commonly accepted conclusions for assessing the toxicity of nanomaterials [29]. Studies have found that mitochondria are the main source of ROS. If the oxidant is imbalanced and expresses antioxidant enzymes and proteins, it will produce oxidative stress [30,31,32]. This condition leads to the formation of peroxides and free radicals, thereby destroying proteins, lipids and DNA, causing a series of cascades such as apoptosis, inflammation and the promotion of tissue and organ damage [33,34]. In our study, ROS content was positively correlated with Ag NPs concentration. When the Ag NPs concentration reached 60 μg/mL, the intracellular ROS content was about twice that of the control group. This indicates that the excessive accumulation of ROS induced by Ag NPs may be an important cause of cytotoxicity.

For detecting the impact of apoptosis-regulating genes such as P53, Bax, Bcl-2 and P21 on the survival or cytotoxicity of HCT116 and NCM460 cells exposed to Ag NPs, we determined the mRNA expression in the cells. In the HCT116 cell line, Ag NPs can promote the increase in P38 protein phosphorylation levels without significant effects on ERK. By promoting the expression of P53 and Bax and the down-regulation of Bcl-2, this leads to an increase in the Bax/Bcl-2 ratio and the activation of P21, which further increases cell death. The difference is that ERK protein phosphorylation is reduced in NCM460 cells. Bcl2 can prevent the release of apoptosis-forming factors (such as cytochrome C) in mitochondria and plays an anti-apoptotic role. Moreover, Bax can act on voltage-dependent ion channels on mitochondria, mediate the release of cytochrome C and promote apoptosis. P53 promotes apoptosis by up-regulating Bax and down-regulating Bcl-2 [35,36]. There have been reports of colorectal cancer cells treated with Ag NPs, and the down-regulation of Bcl-2 expression, which inhibited colon cancer proliferation, has also been recorded [37]. At the same time, the P53 protein activates the transcription and high expression of the P21 gene, stopping the cell cycle at the G1, G2 or S phase and hindering DNA replication and mitosis thereby inhibiting cell proliferation [38,39]. Our flow cytometry results of HCT116 cells can confirm this conclusion (Supplementary Figure S1). Compared with the control, significant concentration-dependent cytotoxicity of the nano Ag in HCT116 cells was observed, in the concentration range of 15 to 60 μg/mL. It has also been found in other related studies that 20 nm Ag particles increase ROS content in LoVo intestinal cell lines, leading to mitochondrial dysfunction and subsequent induced apoptosis [40]. This is similar to our results. However, studies on Ag nano-human liver HepG2 cells and colon Caco2 cytotoxicity studies have shown that the differences in the mechanisms of toxicity induced by nanosilver may be largely a consequence of the type of cells used [41]. This differential, rather than universal, response of different cell types exposed to nanoparticles may play an important role in the mechanism of their toxicity. Similarly, our study also showed that nano Ag has toxic differences on HCT116 and NCM460 cells. In addition, there is no doubt that supplementing the results of studies with molecules such as caspase3 and other cell lines can better prove our experimental conclusions. However, due to the recent severe pneumonia epidemic in China, experimental supplementation of other related genes and cell lines cannot be performed in a short period of time and should be explored further in the subsequent research.

In conclusion, this study evaluated the effect of Ag NPs exposure on colon cells. Ag NPs may induce oxidative stress in cells, increase ROS, reduce cell viability and induce apoptosis. Furthermore, the cytotoxicity is dependent on the concentration of Ag nanoparticles. In summary, these findings provide very useful toxicological information and help to better evaluate the biological safety of nanomaterials.

## 4. Materials and Methods

### 4.1. Nanoparticles

Nano Ag powder was purchased from Beijing Deke Daojin Technology Co., Ltd. (Deke Daojin, Beijing, China). The structure of Ag-NPs was studied by transmission electron microscopy (TEM) JEM-2010 (JEOL Ltd., Tokyo, Japan) and the observation samples were fixed on a copper mesh according to standard procedures. The crystal structure of Ag-NPs was determined by X-ray diffraction (XRD) (BRUKER AXS GMBH, Karlsruhe, Germany) and the particle size was detected by a nanometer particle size analyzer Zetasizer Nano S90 (Malvern Panalytical, Malvern, United Kingdom). Ag-NPs were added to a serum-free 1640 medium before each use and then sonicated for 30 min to fully disperse the nanoparticles.

### 4.2. Cell Culture and Nanoparticle Treatment

A stock solution of Ag NPs (1.0 mg/mL) was prepared in a serum-free RPMI-1640 medium and stored at −20 °C (pay attention to avoid repeated freezing-thawing). The desired concentration (3, 6, 15, 30, and 60 μg/mL) was obtained by diluting the nanoparticle stock solution with a complete cell culture medium each time. The HCT116 and NCM460 cells were provided by the Molecular Nutrition Laboratory of Central South University of Forestry and Technology (Changsha, China). The cells were dispersed in a RPMI-1640 (Invitrogen Gibco, New York, NY, USA) complete medium (containing 10% calf serum and 1% penicillin-streptomycin) and cultured in a 37 °C, 5% CO_2_ incubator for 8 h. The old medium was removed, a fresh medium with different concentrations of nanoparticles was added and incubation was continued for 24 h. The changes to cell morphology under an inverted microscope were observed (Olympus CK 40, Tokyo, Japan) and saved.

### 4.3. Cell Viability Assay

MTT cell proliferation and cytotoxicity assay kits (Sangon, Shanghai, China) were used to detect colon cell viability after exposure to different concentrations of Ag NPs. This method is based on succinate dehydrogenase activity and determines the number of viable cells. The cells (1.0 × 10^5^ mL^−1^) were seeded into a 96-well plate and grown at 37 °C, 5% CO_2_ for adherent growth. The Ag NPs stock solution was added to complete the medium in order to obtain the desired concentration of Ag NPs (3, 6, 15, 30 and 60 µg/mL). The old medium was carefully removed from the 96-well plate and a new medium containing different concentrations of Ag NPs was added and placed in an incubator for further culture. For each concentration, four well replicates were prepared. After 24 h of exposure, 10 μL of MTT solution was added to each cell culture well according to the manufacturer’s instructions. Gently shake the culture plate to evenly disperse the MTT reagent, and continue incubating for 4 h in a 37 °C, 5% CO_2_ incubator. The MTT solution was then discarded, and 100 μL of formazan solution was added to each well, and the plate was gently shaken at room temperature to mix them for 10 min. The optical density (OD) of each well was measured by a multi-functional microplate reader SpectraMax i3x (Molecular Devices, San Jose, California, USA) at a wavelength of 570 nm. Cell viability was calculated as the ratio of the average OD obtained at each nanoparticle concentration to the average OD under control (without Ag nanoparticles).

### 4.4. Intracellular Reactive Oxygen Species (ROS) Assay

Intracellular ROS levels were measured using a fluorescent probe 2′,7′-dichlorodihydrofluorescein diacetate (DCFH-DA) according to the kit manufacturer’s instructions (Beyotime, Shanghai, China). In short, HCT116 cells and NCM460 cells were seeded into 6-well plates with 5 × 10^6^ cells/well and allowed to grow overnight. The cells were then treated with Ag NPs (3, 6, 15, 30 and 60 µg/mL) for 24 h. The cell culture medium was removed, 1 mL of serum-free medium diluted DCFH-DA (10 μM) was added and incubation was continued for 20 min. Finally, the cell culture medium was washed 3 times with PBS to completely remove free DCFH-DA. The fluorescence value of the sample was immediately measured by a multifunctional microplate reader (Molecular Devices, California, USA). The percentage of active oxygen in the cell was calculated by the following formula: test sample fluorescence value/control sample fluorescence value × 100%.

### 4.5. LDH Release

In this study, we used a lactate dehydrogenase (LDH) cytotoxicity assay kit (Beyotime, Shanghai, China) to detect nano-Ag cytotoxicity. LDH is a cytoplasmic enzyme found in all living cells. If cell membranes are damaged and integrity is compromised, intracellular LDH will be released into external media. Therefore, we evaluated the cytotoxicity of nanoparticles by measuring the amount of LDH in the medium after the cells were exposed to nano-Ag. Cells were seeded into 6-well plates (5.0 × 10^6^ cells/well), cultured to adherent growth and then exposed to different concentrations (0, 3, 6, 15, 30, and 60 μg/mL) of Ag nanoparticles. After 24 h, the cell culture plates were centrifuged with a multi-well plate centrifuge at 400 g for 5 min, the supernatant (120 μL) was removed and 120 μL of diluted LDH detection reagent was added. The culture was then mixed and shaken appropriately, and incubation was continued for 1 h. The absorbance of each well was measured using a multifunctional microplate reader (Molecular Devices) at 490 nm. The calculation formula is as follows: cell membrane integrity (%) = absorbance of treated samples/absorbance of sample control wells × 100%.

### 4.6. RNA Isolation and Reverse-Transcriptase PCR Analysis

After cells were exposed to different concentrations of Ag-NP (3, 6, 15, 30, and 60 µg/mL) for 24 h, RNA was extracted using the “Column Total RNA Purification Kit” (Sangon Biotech, Shanghai, China) according to the manufacturer’s instructions. cDNA was synthesized using a M-MuLV First Strand cDNA Synthesis Kit (Sangon Biotech, Shanghai, China) and further detected by RT-PCR using specific primers [42,43]. The PCR conditions include 1 cycle at 95 °C for 3 min, then 40 cycles at 95 °C for 30 s, 60 °C for 30 s and 72 °C for 30 s and, finally, at 72 °C for the extended time of 5 min. The sequences of the specific primers used in this study are shown in Supplementary Table S1. Glyceraldehyde-3-phosphate dehydrogenase (GAPDH) was used as an internal reference gene to quantify the expression level of the target gene.

### 4.7. Western-Blotting Analysis

The cells were seeded in a 10 cm petri dish at a density of 1 × 10^7^ and cultured in a 37 °C, 5% CO_2_ incubator to adhere the cells. The old culture medium was discarded and replaced with a fresh medium containing different concentrations of Ag-NP (3, 6, 15, 30, and 60 μg/mL). Incubation was then continued for 24 h. After the treatment was completed, the cells were washed twice with PBS. The total protein was extracted using a whole protein extraction kit (Sangon Biotech, Shanghai, China) according to the manufacturer’s instructions. 12% sodium lauryl sulfate - polyacrylamide gel (SDS-PAGE) was used to separate the target protein fragment, and then the protein was transferred to a polyvinylidene fluoride (PVDF) membrane. The PVDF membrane was blocked with TBST containing 5% skimmed milk for 30 min at room temperature and then incubated with a specific primary antibody at 4 °C overnight. The PVDF membranes were incubated with horseradish peroxidase (HRP)-conjugated (Sangon Biotech, Shanghai, China) secondary antibodies at room temperature and the blots were developed by a chemiluminescence visualization substrate system. GAPDH were used as a normalization control for equal loading.

### 4.8. Statistical Analysis

The results of at least three independent experiments are presented as mean ± SD. The comparison between the treated and control groups was carried out by ANOVA analysis using the SPSS 23.0 software package (SPSS Company, Chicago, IL, USA) [44]. * *p* < 0.05 was considered as the significance level for all analyses performed.

## Supplementary Materials

Supplementary materials can be found at https://www.mdpi.com/1422-0067/21/5/1618/s1.
