# Supplementary material for: Evaluation of the Genotoxic and Oxidative Damage Potential of Silver Nanoparticles in Human NCM460 and HCT116 Cells
Source: Int J Mol Sci. 2020 Feb 27;21(5):1618. doi: 10.3390/ijms21051618 (PMC7084348; doi:10.3390/ijms21051618)
Supplement: Supplementary file 1 [file ijms-21-01618-s001.pdf]

International Journal of
Molecular Sciences

Supplementary Materials:

Evaluation of the Genotoxic and Oxidative Damage Potential of Silver Nanoparticles in Human NCM460 and HCT116 Cells

Mingxi Jia [1,2,†], Wenjing Zhang [1], Taojin He [1], Meng Shu [1], Jing Deng [1,2,†], Jianhui Wang [3,†], Wen Li [1,2,*], Jie Bai [1,*], Qinlu Lin [1], Feijun Luo [1], Wenhua Zhou [1] and Xiaoxi Zeng [2]

Table S1. Gene Specific Primers Used for RT-PCR.

Gene name	Primer Sequence (5′–3′)
GAPDH Forward	CAGGAGGCATTGCTGATGAT
GAPDH Reverse	GAAGGCTGGGGCTCATTT
P53 Forward	TCAGTCTACCTCCCGCCATA
P53 Reverse	TTACATCTCCCAAACATCCCT
Bax Forward	ACGAACTGGACAGTAACATGGAG
Bax Reverse	AGTTTGCTGGCAAAGTAGAAAAG
Bcl2 Forward	CATGGCAGTAAAGCAAG
Bcl2 Reverse	ATTGTTCCCATAGAGTTCCACAA
P21 Forward	ATGTGGACCTGTCACTGTCTTGTA
P21 Reverse	GTTGGAGTGGTAGAAATCTGTCAT

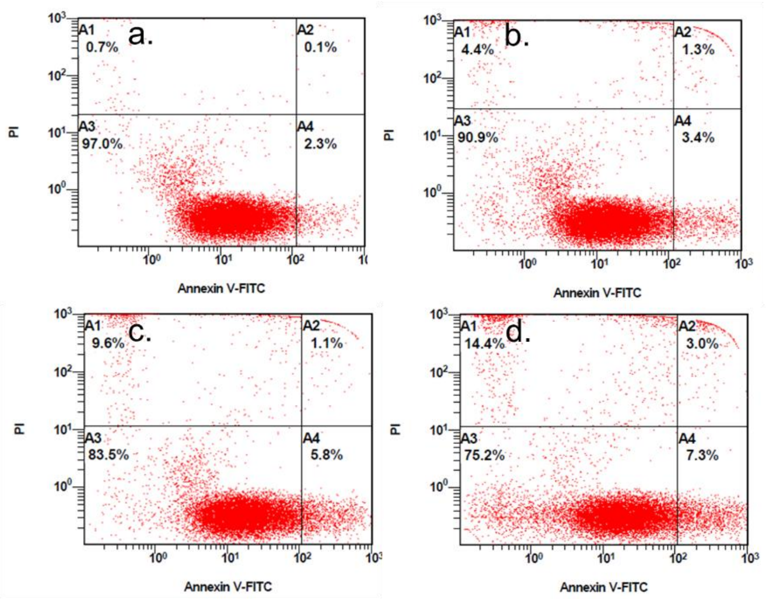

Figure S1. Flow cytometry results of HCT116 cells exposed to Ag nanoparticles. (**a**) control group; (**b**) 15μg/mL group; (**c**) 30 μg/mL group; (**d**) 60 μg/mL group.
